# Geoffrey C Schild (1935‐2017)

**DOI:** 10.1111/irv.12484

**Published:** 2017-11-28

**Authors:** John Wood, Alan Hay

**Affiliations:** ^1^ National Institute for Biological Standards and Control Potters Bar Herts UK; ^2^ WHO Collaborating Centre for Reference and Research on Influenza Visiting Scientist The Francis Crick Institute London UK

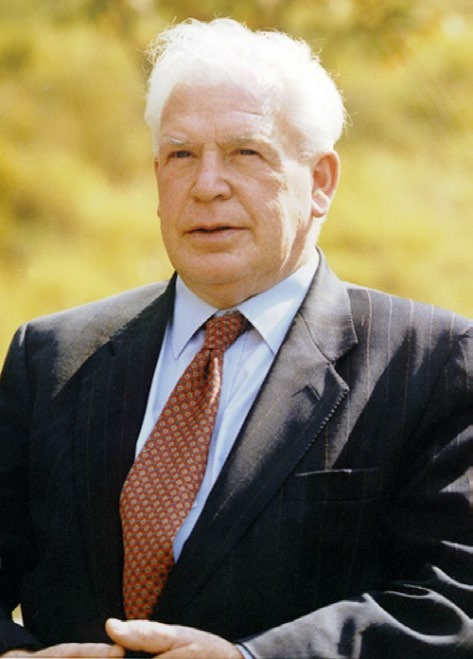

On 3 August 2017, Dr Geoffrey Schild passed away after battling with illness for several years. Geoffrey was a world‐renowned virologist who had a major influence in the fields of influenza virus surveillance and prevention, poliovirus eradication, AIDS vaccine development and the virological safety of blood products.

He began his scientific career in the department of Sir Charles Stuart‐Harris at the University of Sheffield in 1960, working on influenza, polio‐ and rhinoviruses. His early interest in influenza viruses was kindled by work on serological responses to influenza infection, which formed a basis for much of his subsequent contributions to our understanding of influenza and efforts for its control. Geoffrey then joined the scientific staff of the Medical Research Council (MRC) National Institute for Medical Research (NIMR), Mill Hill, London (1967‐1975), where he continued to work on influenza viruses and from 1969 to 1975 served as Director of the World Health Organization (WHO) World Influenza Centre. During his time at the NIMR, he was instrumental in developing the system for classification of influenza viruses based on antigenic subtypes of haemagglutinin and neuraminidase,^1^ and in developing the single‐radial‐immunodiffusion (SRID) influenza vaccine potency assay 2 that remains the international gold standard.

His interests broadened on his move in 1975 to the National Institute for Biological Standards and Control (NIBSC), Hampstead, UK, as Head of the Viral Products Division. As Director of the Institute, from 1985 till his retirement in 2002, he oversaw its move to South Mimms in 1987 and ushered in a new era in the development of the scientific basis for quality and safety of biologicals, with the provision of many key WHO international standards and reference materials for quality control of bacterial and viral vaccines and other immunologicals. Notable achievements in the 1980s and 1990s were as follows: reform of safety testing of blood and blood products throughout the world by the introduction of reference materials,^3^ such that virological contamination became a rare event; direction of the UK MRC AIDS Directed Programme (1987‐1992)^4^; direction of poliovirus research, first to rationalise the test for vaccine virus attenuation,^5^, ^6^ then to better understand virus variation and immune recognition and finally to develop new vaccine strategies, all of which have been crucial to the polio eradication programme; direction of the regulation of biotechnological products in the EU, chairing the first EU Ad Hoc Working Party on Biotechnology.^7^

Geoffrey was an influential participant in the annual WHO consultations on influenza vaccine composition and was the instigator of the associated vaccine manufacturers meetings that he chaired for many years.

Geoffrey was a founder member, the first Chair and the inspiration behind the formation of the International Society for Influenza and other Respiratory Virus Diseases in 2005. He also served as the first Editor in Chief of *Influenza and other Respiratory Viruses* in 2007. He became an honorary Vice President of isirv and in 2016 received from isirv an “Extraordinary Award for a lifetime of Research in Influenza.”

During his long and distinguished career, Geoffrey was an inventor, a pioneer and a natural leader with enormous insight and vision. He was an inspiration to his many colleagues and to young scientists, was always enthusiastic about their interests, encouraging their research. Geoffrey's contribution to science has been recognised by fellowships of many prestigious professional societies, and in 1993, he was awarded the honour of Commander of the British Empire (CBE) for Services to Science.

Working with Geoffrey was never dull. He had his own style of organised chaos, which could infuriate or amuse depending on your point of view. His long‐time friend and colleague Professor Ian Gust commented “Geoffrey was an unforgettable presence, filling the room or restaurant with his personality and voice, generating ideas and challenging assumptions with a chuckle and a wicked grin.” Geoffrey was a “one‐off,” a larger‐than‐life character, charismatic and generous. He had a passion for the correct use of English, vigorously editing and improving many WHO documents. His prose and phrases are legendary.

On retirement, he enjoyed a brief consultancy on plant‐based vaccines and afterwards lived with his wife Tora in her beautiful family home north of Bergen, Norway, surrounded by his family (three children, a grandchild and countless nephews and nieces). Here he could enjoy his passion for nature, especially birdwatching.

He will be missed but not forgotten.
